# Behavioral, emotional and social functioning in children born with congenital diaphragmatic hernia

**DOI:** 10.1007/s00383-018-4266-9

**Published:** 2018-04-10

**Authors:** Elin Öst, Margret Nisell, Carmen Mesas Burgos, Björn Frenckner, Maria Öjmyr-Joelsson

**Affiliations:** 1 Department of Women’s and Children’s Health, Karolinska Institutet, 171 76 Stockholm, Sweden; 2 Pediatric Surgery Unit, Karolinska University Hospital, Astrid Lindgren Children’s Hospital, 171 76 Stockholm, Sweden; 3 The Red Cross University College, 141 52 Huddinge, Sweden

**Keywords:** Child behavior check-list, Psychosocial function, Congenital diaphragmatic hernia, Long-term follow-up, Extracorporeal membrane oxygenation

## Abstract

**Purpose:**

The aim was to investigate social competence and behavioral and emotional problems in children and adolescents born with CDH.

**Methods:**

All children born with CDH, treated in Stockholm 1990–2009, were invited to participate. After written consent, the Child Behavior Checklist or Adult Self-Report questionnaires were sent to participants. Of the 145 long-term survivors, 51% returned a completed questionnaire. Both the syndrome and competence scales were used and open-ended questions were analyzed with manifest content analysis.

**Results:**

All parents of children aged 1.5–5 years and 90% of parents of children aged 6–18 years reported a normal range on the syndrome scale. Five parents indicated internalizing, but none externalizing behavior. All young adults achieved a normal score on the syndrome scale. Eighty-five percent had normal school achievement, 79% had normal social scores and 40% had normal activity levels. Significantly fewer boys (23%) were in the normal activity range compared with 67% of girls.

**Conclusions:**

The vast majority of all parents of children born with CDH scored no behavioral or emotional problems, furthermore, they reported normal social and school competence. However, the activity levels seemed to be reduced in children born with CDH.

## Background

Congenital diaphragmatic hernia (CDH) is a rare malformation with a still unknown etiology [1]. The severity of the malformation varies and this can be reflected in the variation in length of hospital stay (LOS) which, itself, seems to be dependent on the size of the diaphragmatic defect [2]. While some children recover after a few weeks in hospital, others stay for many months and extra corporeal membrane oxygenation (ECMO) is sometimes required. Progress in intensive care treatments over the last decade has led to an increased survival rate for children born with CDH [3, 4], and children with a severe form of the malformation most likely represent a new group of survivors. Observations from long-term follow-ups of children with CDH have shown associated morbidity in a large part of the group, with pulmonary-, gastrointestinal-, neurodevelopmental- and musculoskeletal-related symptoms [5, 6]. Again, the size of the diaphragmatic defect seems to be the greatest predictor of overall morbidity at discharge [2]. Severely ill neonates have demonstrated negative psychological and behavioral outcomes later in life [7] and the long-term follow-up of CDH survivors has, over the last few years, gained more attention. Additionally, the interest in measuring outcomes beyond mortality and morbidity has increased. There are, however, still currently few studies on the subject.

We hypothesized that children born with CDH who were severely ill during the neonatal period might suffer from adverse psychological and behavioral issues later in life. The aim of this study was to study social competence and behavioral and emotional problems in children born with CDH and, furthermore, investigate possible risk factors for these behaviors within this cohort.

## Methods

### The parents and their children

Between 1990 and 2009 a total of 185 newborn children with CDH were treated in Stockholm, Sweden. The primary survival rate, when discharged from hospital either to their homes or to a hospital closer to home, was 85% (157 children). Due to the Swedish personal identification number (PIN) all the children could be tracked, and in 2012 there were 145 long-term survivors (78%). All the long-term survivors were asked to participate in the study. Seventy-four parents or adolescents of majority age returned a completed questionnaire. The response rate was 51%.

Data on gender, prenatal diagnosis, birth weight, gestational age, side of lesion, method of surgical repair, time to intubation, history of ECMO treatment, and type of discharge from hospital were collected from the case records for all the patients during the time period.

### Questionnaires

We used the Child Behavior Checklist (CBCL), which is a questionnaire designed to assess competences and behavioral and emotional problems in children and adolescents [8]. There are different versions depending on the age of the child or adolescent. There is one CBCL version for children aged 1.5–5 years and another for older children (CBCL 6–18 years). Both these questionnaire versions are answered by parents or others who know the children in their normal home settings. For young adults > 18 years of age, the Adult Self-Report (ASR) was used which is a continuation of the CBCL but adapted to a more adult way of life and is answered by the individuals themselves. In common for the three questionnaires is the syndrome scale with 100–126 statements of behavioral, emotional and social problems based on the previous 2 months for the youngest population and the last 6 months for children and adolescents older than 6 years of age. Responses are rated as 0 = not true (as far as you know), 1 = somewhat or sometimes true, and 2 = very true or often true. The answers are added to subgroups and the responses from these are then analyzed according to a specific profile sheet. The profile sheet shows what is, or what is not, classified as normal and the answers also generate a *T*-score. Since previous published articles describe the results in both *T*- and raw scores, both of these will be presented. For the syndrome scale, a *T*-score < 65 is normal, > 70 is clinical, and in-between is borderline. Answers from the syndrome scale generate a total value, in addition to internalizing or externalizing behavior, which is age-adjusted. An internalizing behavior in children aged 1.5–5 years includes withdrawn, somatic complaints, anxious/depressed and emotionally reactive behavior, while an externalizing behavior includes attention problems and aggressive behavior. For children 6–18 years an internalizing behavior includes withdrawn, somatic complaints, anxious/depressed, and an externalizing behavior includes rule-breaking- and aggressive behavior. In young adults an internalizing behavior is described as anxious/depressed, withdrawn, somatic complaints and an externalizing behavior as aggressive-, rule-breaking and intrusive behavior.

Both the CBCL questionnaires end with three open-ended questions where the parent can describe: (1) the child’s illnesses and disabilities, (2) concerns about the child and, (3) the best things about the child. On the ASR the young adult is asked if he/she has: (1) any illness, disability, or handicap; (2) concerns or worries about family, work, education or anything else and; (3) to describe the best things about him/herself.

For the CBCL 6–18, the competence scale was also used, divided into four aspects: activities, social relations, school, and total competence [8]. Regarding the competence scale, a *T*-score > 35 is normal, < 30 is clinical, and in-between is borderline.

### Ethics

This study was approved by the regional ethical committee in Stockholm, Dnr 2011/472-31/4. Written informed consent was obtained upon inclusion in the study from the parents of underage children and adolescents of majority age.

### Data analysis

A Chi-square test was used to analyze differences between the study population, the entire group and non-participants. The three different questionnaires were analyzed separately according to the handbooks for the CBCL 1.5–5, CBCL 6–18 and ASR [8–10]. A total problem score, sub-scores and two broad-band groupings of behavioral, emotional and social problems (internalizing and externalizing) were calculated. The total problem, internalizing and externalizing scores were then converted into *T*-scores according to profiles where normal and clinical range are given. Fisher’s exact test was used to analyze categorical differences between the sub-groups according to gender, age at intubation, ECMO support, and side of the hernia. Spearman’s correlation test was applied to analyze the correlation between CBCL values and LOS.

The open-ended questions were analyzed with manifest content analysis inspired by Graneheim and Lundman [11]. All the answers were carefully read and written down. The text was then condensed into meaning units consisting of words that were reminiscent of the original text. The final step was to code the condensed meaning units into sub-categories and categories.

## Results

### Participant and patient characteristics

A total number of 74 questionnaires were analyzed. Fifteen (65%) parents answered the CBCL 1.5–5, 48 (50%) parents answered the CBCL 6–18, and 11 (42%) young adults answered the ASR. The participants did not differ from the entire cohort nor the non-participants who had declined to participate or had been excluded from the study regarding prenatal diagnosis, gender, side of lesion, method of surgical repair, time to intubation, need for ECMO support or type of hospital discharge. There were, however, several significant differences between the participants and children who had died, since the latter group represents the most severely affected children. Furthermore, prenatal diagnosis, immediate intubation, need for ECMO support, patch repair, and right-sided hernias were more frequent in the group of deceased children (for further characteristics see Table 1). Length of hospital stay (mean of 44 days, range 5–304 days) was measured within the group of participants to see possible correlations with the results from the CBCL.

**Table 1:** Demographic data for all children with CDH treated at Astrid Lindgren Children’s hospital 1990–2009 (both children of study participants and non-particpants) *n* (%)

	Entire cohort *n* = 185	Study participants *n* = 74	Non-participants *n* = 111	
			Declined or excluded *n* = 71	Deceased *n* = 40
Gender				
Male	118 (64)	45 (61)	43 (61)	30 (75)
Female	67 (36)	29 (39)	28 (39)	10 (25)
Prenatal diagnosis	67 (36)	24 (32)	18 (25)	25 (63)*
Birth weight (kg) (mean ± SD)	3.3 ± 0.7	3.5 ± 0.6	3.2 ± 0.6	3.1 ± 0.8
Gestational age (weeks) (mean ± SD)	38.3 ± 2.4	38.8 ± 2.2	38.4 ± 2.5	37.2 ± 2.2
Side of lesion				
Left	145 (78)	65 (88)	57 (80)	23 (58)*
Right	22 (12)	7 (9)	10 (14)	5 (13)
Bilateral	1 (0.5)	0 (0)	0 (0)	1 (2.5)
Repaired				
Primary	98 (53)	44 (59)	49 (69)	6 (15)*
Patch	65 (35)	27 (36)	17 (24)	20 (50)
Intubated within 6 h from birth	134 (72)	48 (65)	48 (68)	37 (93)*
ECMO	54 (29)	18 (24)	12 (17)	24 (60)*
ECMO > once	13 (7)	3 (4)	4 (6)	6 (15)
Referred to other hospital	49 (26)	21 (28)	22 (31)	6 (15)
Survival to discharge	157 (85)	74 (100)*	71 (100)	12 (30)*
Long-term survivors (2012)	145 (78)	74 (100)*	71 (100)	0 (0)*

*p < 0.05, when compared with study participants

### Quantitative results from the child behavior checklist

**Fig. 1:**
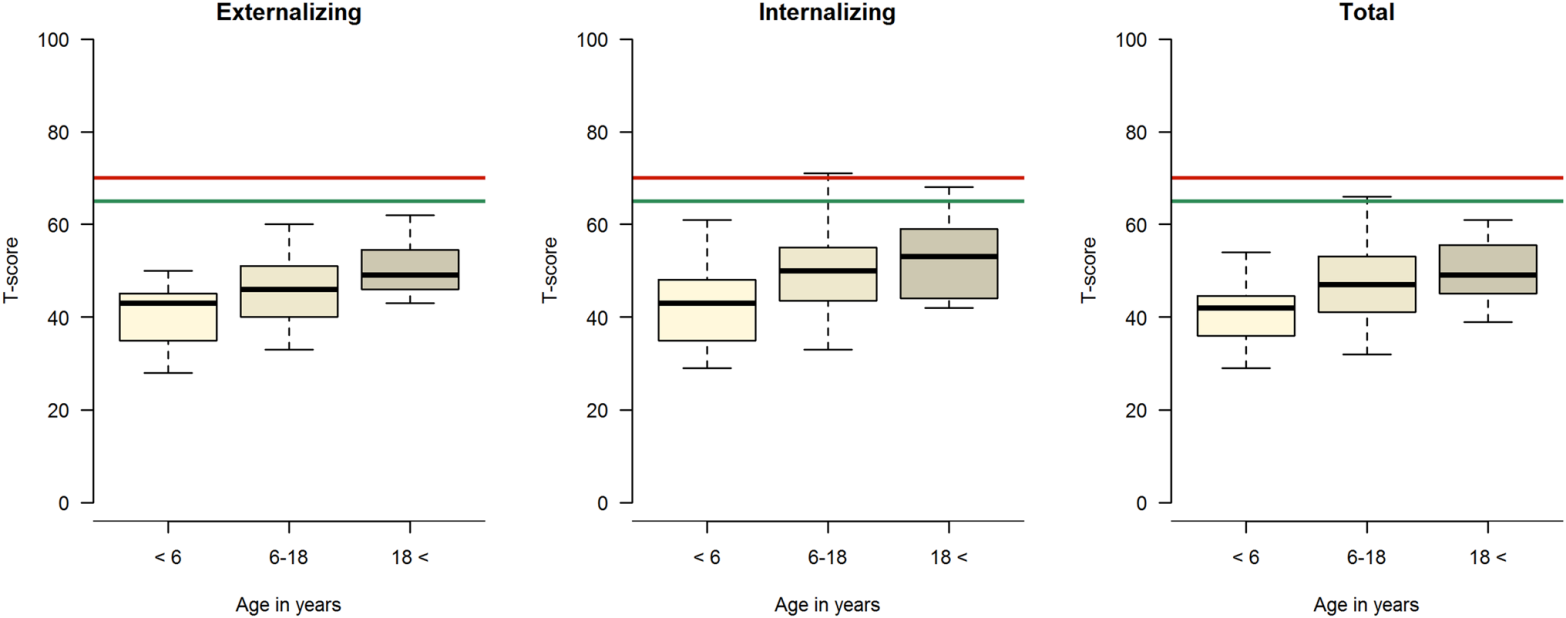
CBCL and ASR Syndrome scale. *T*-scores regarding externalizing and internalizing behavior and total in different age groups. The thick line in the box represents the median, the box represents the interquartile range, the bars represent the upper- and lower quartiles. Values below the green line are normal-, above the red line clinical- and in between borderline range

For descriptive data concerning quantitative results for the CBCL 1.5–5, CBCL 6–18 and ASR, please see Fig. 1 and Table 2.

**Table 2:** CBCL and ASR raw scores syndrome scale mean (SD)

	CBCL 1.5–5 *N* = 15	CBCL 6–18 *n* = 48	ASR *N* = 11
Problem areas			
Emotionally reactive	1.6 (1.8)	–	–
Anxiety/depression	0.7 (1.1)	2.2 (2.4)	7.3 (5.4)
Withdrawn/depressed	0.6 (0.6)	1.4 (1.7)	2.4 (2.1)
Somatic complaints	1.6 (1.5)	1.9 (2.5)	3.3 (2.6)
Sleep problems	1.7 (2.3)	–	–
Social problems	–	1.6 (2.4)	–
Thought problems	–	1.0 (1.7)	2.2 (1.4)
Attention problems	1.1 (0.9)	2.4 (2.9)	6.2 (3.2)
Rule-breaking behavior	–	0.9 (1.3)	1.9 (1.6)
Aggressive behavior	4.5 (3.6)	2.9 (2.9)	5.1 (3.8)
Intrusive	–	–	2.3 (1.9)
Other problems	5.2 (3.8)	2.3 (2.4)	9.6 (4.7)
Internalizing	1.1 (1.4)	1.8 (2.3)	4.3 (4.2)
Externalizing	2.8 (3.1)	1.9 (2.5)	3.1 (2.9)
Total problem	17.1 (10.7)	16.6 (13.6)	40.2 (18.6)

All the parents of children aged 1.5–5 years scored within a normal range on the syndrome scale. One parent reported sleeping problems. Six of the parents of the youngest children reported that their child had a disease or some kind of remaining handicap where impaired lung function was mentioned three times and other associated impairments three times.

Forty-three parents (90%) in the group of children 6–18 years of age scored within a normal range on the syndrome scale, while five parents scored within a borderline range. A total of five parents indicated internalizing behavior, whereas three were considered as borderline and two clinical. None were in the externalizing range. Parents reported somatic complaints in four children. Significantly more children who had received ECMO support had internalizing behavior. There were no differences on the syndrome scale regarding gender, side of hernia or time to intubation (Table 3). Of the parents of children aged 6–18 years, twelve (25%) mentioned a disease or some kind of handicap where impaired lung function and asthma occurred seven times, developmental delay three times, neuropsychiatric disorder once and another syndrome once.

**Table 3:** CBCL 6–18 comparisons between groups, numbers of children within different categories

	All *n* = 48/34	Intub < 6 h *n* = 30/19	Intub > 6 h *n* = 15/12	ECMO *n* = 9/5	No ECMO *n* = 39/29	Boys *n* = 30/20	Girls *n* = 18/14	Left *n* = 41/30	Right *n* = 5/4
Social competence									
Activity									
Normal	19 (40)	11 (37)	6 (40)	2 (22)	17 (44)	7 (23)*	12 (67)	16 (39)	1 (20)
Borderline	16 (33)	11 (37)	4 (27)	4 (44)	12 (26)	11 (37)	5 (28)	14 (34)	2 (40)
Clinical	13 (27)	8 (27)	5 (33)	3 (33)	10 (31)	12 (40)*	1 (6)	11 (27)	2 (40)
Social									
Normal	38 (79)	22 (73)	13 (87)	4 (44)*	34 (87)	22 (73)	16 (89)	34 (83)	2 (40)
Borderline	4 (8)	3 (10)	1 (7)	2 (22)	2 (5)	3 (10)	1 (6)	3 (7)	1 (20)
Clinical	6 (13)	5 (17)	1 (7)	3 (33)	3 (8)	5 (17)	1 (6)	4 (10)	2 (40)
School									
Normal	29 (85)	17 (89)	10 (83)	3 (60)	26 (90)	18 (90)	11 (79)	26 (87)	2 (50)
Borderline	2 (6)	1 (5)	0	1 (20)	1 (3)	0	2 (14)	2 (7)	1 (25)
Clinical	3 (9)	1 (5)	2 (17)	1 (20)	2 (7)	2 (10)	1 (7)	2 (7)	1 (25)
Total scores									
Normal	22 (65)	10 (53)	10 (83)	2 (40)	20 (69)	12 (60)	10 (71)	21 (70)	0*
Borderline	8 (24)	7 (37)*	0	1 (10)	7 (24)	5 (25)	3 (21)	7 (23)	2 (50)
Clinical	4 (12)	2 (11)	2 (17)	2 (40)	2 (7)	3 (15)	1 (7)	2 (7)	2 (50)
Syndrome scale									
Internalizing									
Normal	43 (90)	27 (90)	13 (87)	6 (67)*	37 (95)	27 (90)	17 (94)	38 (93)	4 (80)
Borderline	3 (6)	2 (7)	1 (7)	2 (22)	1 (2,5)	2 (7)	1 (6)	1 (2)	1 (20)
Clinical	2 (4)	1 (3)	1 (7)	1 (11)	1 (2,5)	1 (3)	0	2 (5)	0
Externalizing									
Normal	48 (100)	30 (100)	15 (100)	9 (100)	39 (100)	30 (100)	18 (100)	41 (100)	5 (100)
Borderline	0	0	0	0	0	0	0	0	0
Clinical	0	0	0	0	0	0	0	0	0
Total problem									
Normal	43 (90)	29 (97)	13 (87)	8 (89)	35 (90)	26 (87)	17 (94)	37 (90)	5 (100)
Borderline	5 (10)	1 (3)	2 (13)	1 (11)	4 (10)	4 (13)	1 (6)	4 (10)	0
Clinical	0	0	0	0	0	0	0	0	0

Number of patients given in each group and percentage within brackets

*p < 0.05 comparisons between groups, Fisher’s Exact Test

In the group of young adults, everyone scored within a normal range on the syndrome scale. One person had internalizing borderline behavior, while no one in this group had externalizing behavior. Of the young adults, 27% had remaining disease or a handicap in terms of asthma, mild developmental delay, or difficulty with gaining weight.

**Fig. 2:**
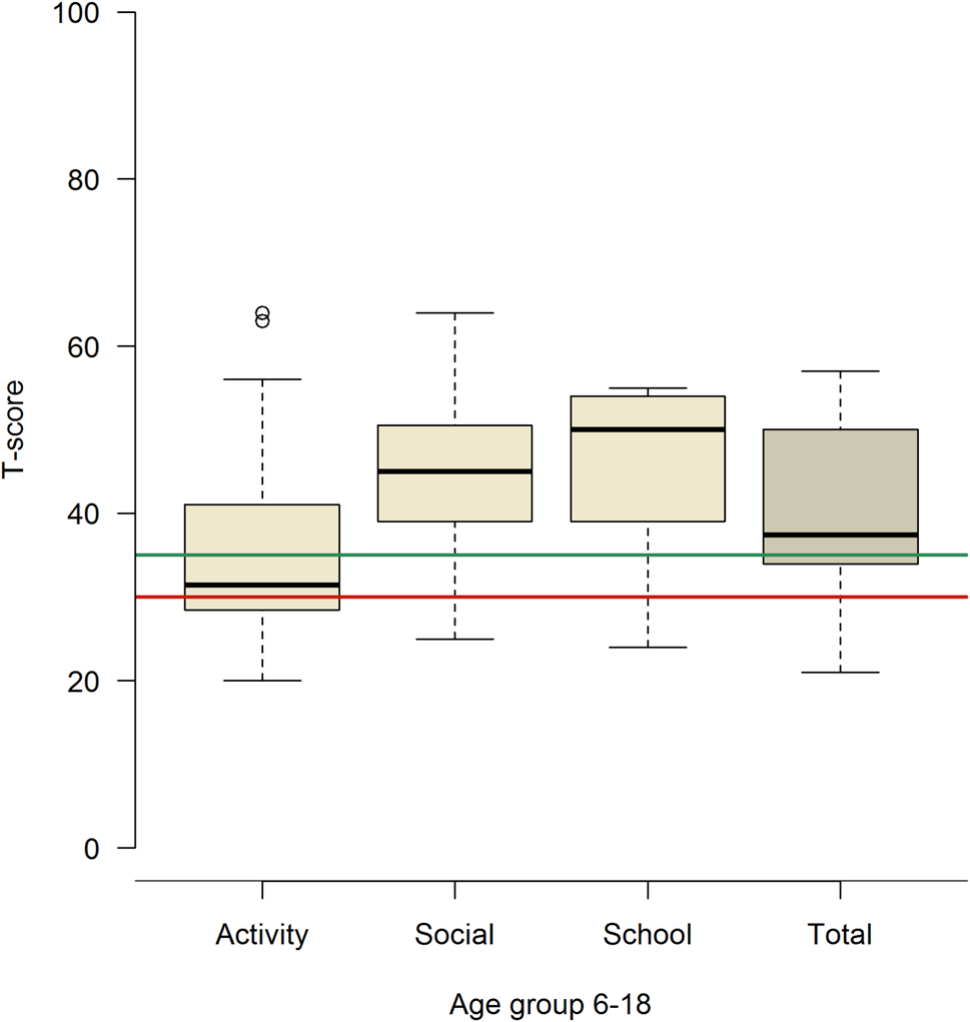
Competence scale *T*-scores, children 6–18 years. The thick line in the box represents the median, the box represents the interquartile range, the bars represent the upper and lower quartiles. Values above the green line are normal-, below the red line clinical- and in between borderline range

Regarding the competence scale, children 6–18 years of age who had not yet started school (*n* = 14) were excluded from the school-related questions in addition to the total scores on the competence scales since school achievements are included. Twenty-two (65%) parents registered a normal score on the total competence scale, while four were within the clinical range and eight borderline. Forty percent ended up within a normal activity level, while 27% had below the clinical activity range and the remaining 33% in-between. Seventy-nine percent had a normal social score, while 13% ended up below the clinical range and the remaining 8% were borderline. Normal school scores were achieved in 85% of the children, while 9% were clinical and 6% borderline (Fig. 2). There were a significantly lower number of boys (*p* = 0.003) with a normal activity score compared with girls and, furthermore, a significantly larger number (*p* = 0.008) of boys scored within the clinical range. None of the children with right-sided diaphragmatic hernia achieved normal total scores (*p* = 0.05). The total score on the competence scale was significantly lower in children who had been intubated within their first 6 h of life. Fewer children who had received ECMO support had normal social scores (*p* = 0.01) (Table 3).

### Qualitative results from the child behavior check-list

#### Child behavior check-list 1.5–5

Analysis of the answers from the open-ended question regarding the parents’ concerns about their children resulted in three categories. The first category was sequelae of CDH, most often related to lung function where respiratory tract infections were a big concern. The second concern was social issues, such as being an outsider. The third category was a general concern that something would happen to their child. Three categories emanated from the analysis about the child’s best quality; social skills, competence and “fighter”. Which involved being empathetic, social, intelligent, and a strong individual with strong vitality.

#### Child behavior check-list 6–18

Two categories emerged regarding the parents’ concerns about their children; future options, i.e., they would not be able to meet the requirements that would be put on them in the future and the effects of having poor lung function and growth. The second concern was about their child’s self-esteem, often related to scars or other defects such as chest wall deformities. Two categories appeared from the child’s best quality; the first one was social skills where the child was empathetic, social, kind and reliable. The second category was competence, which involved being creative, curious, alert and intelligent.

#### Adult self-report

Three categories emanated from the answers from the young adults. The first category was a general concern involving economy, school achievements and family. The second category was social skills which comprised being a good friend, thoughtful and social. The third category was competence. Please see Table 4 for an outline of the manifest content analysis.

**Table 4:** Manifest content analysis open-ended questions

Sub-category	Category
Concerns about child 1.5–5 years *n* = 12 (80%)	
Lung function	Sequelae
Respiratory tract infections	Sequelae
Eating problems	Sequelae
Being outside	Social issues
Interaction with others	Social issues
Something might happen	General concern
Best thing about child 1.5–5 years *n* = 15 (100%)	
Empathetic	Social skills
Social	Social skills
Intelligent	Competent
Strong individual	Fighter
Strong vitality	Fighter
Concerns about child 6–18 years *n* = 28 (58%)	
Meet requirements	Future options
Effects of poor lung function	Future options
Poor growth	Future options
Scars	Self-esteem
Chest-wall deformities	Self-esteem
Best thing about child 6–18 years *n* = 45 (94%)	
Empathetic	Social skills
Social	Social skills
Kind	Social skills
Reliable	Social skills
Creative	Competent
Curious	Competent
Alert	Competent
Intelligent	Competent
Concerns about oneself > 18 years *n* = 4 (36%)	
Economy	General concern
School achievements	General concern
Family	General concern
Best thing about oneself > 18 years *n* = 11 (100%)	
Good friend	Social skills
Thoughtful	Social skills
Social	Social skills
Ambitious	Competent

## Discussion

In this study, we found that parents of children born with CDH scored within the normal range on the behavioral, emotional, and social scales. Furthermore, children born with CDH seemed, according to their parents, to have normal levels of school and social competence. There were, however, less than half within the group who were reported to have an activity level within the normal range.

There are a few other studies of CBCL in children born with CDH that have, however, shown varying results. Peetsold et al. described that 21% of children born with CDH had total problem scores in the clinical range compared with the 10% of their reference population [12], whereas in our present study, none was in the clinical range and 10% of the children aged 6–18 years were in the borderline range. Similar to our study, Peetsold et al. did not see any correlation with the severity of the malformation [12]. Madderom et al. found that 26% of CDH survivors were within the clinical to borderline range regarding the total scale, and 26% on the internalizing and 15% on the externalizing scale. These results, however, were not significantly different from their reference population [13] which is in contrast to the 10% of children 6–18 years old and 9% of the young adults with an internalizing behavior in our study. Additionally, Madderom et al. did not see any significant differences between children with or without ECMO support [13], while significantly more children with a history of ECMO support in our study had an internalizing behavior in the 6–18-year-old group. An internalizing behavior during the period of 6–18 years of age is based on anxiety and withdrawn and somatic complaints without a specific medical reason. In a study of 5-year-old children with a previous history of ECMO support for different reasons (meconium aspiration syndrome, CDH, sepsis or persistent pulmonary hypertension), CBCL scores did not differ from the reference population [14]. In our study, there were 18 children with a history of ECMO support and nine of the children were within the 6–18 year age group. The group as a whole showed large variations and although the differences were significant, it is probably wise to interpret the results with caution because they refer to a small sample size.

In contrast to our study, Bouman et al. found in a small CDH cohort consisting of 11 children, increased levels of emotional and behavioral problems compared with the general population and recommended an intense follow-up to detect children with psychological and social problems [15].

Kubota et al. studied psychological consequences after major neonatal surgery including children born with CDH, anorectal malformations and esophageal atresia, and found higher total *T*-scores in children who underwent multiple surgery and home medical treatment than in those who underwent single surgery without home medical treatment [16]. Early exposure to anesthesia and surgery has previously been identified as significant independent risk factors for learning disabilities but not, however, for emotional or behavioral outcomes [17]. Kubota et al. did not see any correlations with CBCL and LOS scores, which is in line with our results. The total *T*-score in the CDH population in the study by Kubota et al. was 55.3 ± 10.8 [16], while children at the same age in our study had a total *T*-score of 47.2 ± 8.5. In several previous comparisons of the CBCL 6–18 in different societies, it has been found that Swedish and Scandinavian norm data are significantly lower than many other countries [18–20]. It has not yet, however, been explained why the Swedish parents score lower.

In an earlier published article, where data for the Swedish normal population of the older CBCL version concerning preschool children are described [21], scores were much lower than in other parts of the world, but similar to other Scandinavian countries. Even with the new CBCL version, the lower Scandinavian scores have been confirmed in an international comparison of preschool children’s behavioral and emotional problems [20]. Since no new Swedish standard data are available after changing the CBCL questionnaires, there is no possibility to make a comparison with the Swedish normal population. However, for the 1.5–5 year age group, new national Danish normative data are available, which, according to Table 5, are consistent with data from this study [22].

**Table 5:** Total problem syndrome scale raw scores CBCL 1.5–5 years

	Danish normative data	Study population
Total		
Boys and girls	17.3	17.1
Boys	17.5	16.4
Girls	16.7	18.4
Internalizing		
Boys and girls	3.9	4.5
Boys	3.8	4.9
Girls	4.0	3.8
Externalizing		
Boys and girls	6.7	5.7
Boys	6.8	5.6
Girls	6.5	5.8

In our study we found large differences in the activity scale among boys (6.2 ± 2.8) and girls (8.4 ± 3.3) born with CDH aged 6–18 years. In a published study from Norway, Jozefiak et al. presented Norwegian normative data on the CBCL, which has been confirmed as being representative to Swedish normative data [18] (Table 6). Further, Jozefiak et al. showed similar activity in both boys and girls (9.2 ± 2.7), which is much higher than in our study population. The underlying reasons for the low activity rates among children and adolescents born with CDH have not been further elucidated in this study, however, an explanation may be the reduced lung function that many children born with CDH live with. There is, of course, no reason to believe that boys would have a worse lung function than girls but, on the other hand, there are traditional differences between sports that boys and girls often exercise. A speculation could be that it is easier for girls to find traditional sports to exercise that do not require as much physical effort.

**Table 6:** CBCL 6–18 raw scores comparisons of mean (SD) between different populations

	CDH 6–18	Norwegian norm scores	No ECMO
Social competence			
Activity	7.0 (3.2)	9.2 (2.7)	7.3 (3.0)
Social	7.4 (2.6)	8.8 (1.9)	7.9 (2.3)
School	4.7 (1.3)	4.8 (0.9)	4.8 (1.1)
Total scores	20.3 (5.0)	22.9 (4.1)	21.0 (4.6)
Problem areas			
Anxiety/depression	2.2 (2.4)	1.9 (2.5)	1.9 (1.9)
Withdrawn/depressed	1.4 (1.7)	1.0 (1.5)	1.3 (1.6)
Somatic complaints	1.9 (2.5)	1.3 (1.7)	1.7 (2.2)
Social problems	1.6 (2.4)	1.3 (2.0)	1.4 (2.3)
Thought problems	1.0 (1.7)	0.8 (1.3)	0.9 (1.4)
Attention problems	2.4 (2.9)	2.4 (2.7)	1.9 (2.4)
Rule-breaking behavior	0.9 (1.3)	1.1 (1.8)	0.8 (1.4)
Aggressive behavior	2.9 (2.9)	2.7 (3.7)	2.8 (2.8)
Other problems	2.3 (2.4)	–	2.0 (2.3)
Internalizing	1.8 (2.3)	4.2 (4.7)	1.6 (1.9)
Externalizing	1.9 (2.5)	3.8 (5.1)	1.8 (2.4)
Total problem	16.6 (13.6)	14.2 (14.1)	14.7 (12.8)

The findings from the qualitative data showed that there was a clear trend in what worried parents of children of different ages. Parents of the youngest children were very concerned about complications related to CDH and respiratory tract infections and, furthermore, they were generally worried that something would happen to their children. Parents of older children were more concerned about how their children would cope in the future. One possible cause of this shift in anxiety could be that the first few years may have passed by relatively well, and even though the children had respiratory tract infections the parents were able to cope and focus more on the future. Overall, the parents who expressed concerns were those who had children with some kind of sequelae and, therefore, it was a natural reaction. The number of parents who answered the question about being concerned were among the younger group of 80%, while 58% of the parents of the older children did the same, which also reinforces this reflection.

The young children’s parents used strikingly emotional words when describing their children, “she has survived against all the odds” and “he is a real fighter, simply amazing”. Most children born with CDH undergo a very intensive first period of life, and these memories are likely to remain strong among the parents. Even parents of the older children left strong descriptions of their children’s best episodes, although no longer with words like “survivors”.

### Strengths and limitations

The response rate in this study was 51% and even though no significant differences could be shown between participants and non-participants this still has to be taken into account. Comprehensive assessments from different individuals around the child or adolescent are desirable to obtain diverse aspects of adaptive functioning to capture strengths and needs. One limitation of this study is that we only asked parents about their views of their child. It would have been interesting to compare those results from the older children’s own perceptions of themselves, but additionally, teachers could also have been included. In an earlier study from our group regarding health-related quality of life (HRQoL) in children and adolescents born with CDH [23], we found correlations between answers from children and their parents to be very low. In this study, we matched parental CBCL results with results from the study on HRQoL on similar domains/scales, and found strong associations between the answers. Since the results of the CBCL have previously been shown to differ depending on who completed the questionnaire, there is reason to believe that the results would appear different if the children had completed it.

## Conclusion

The vast majority of all children born with congenital diaphragmatic hernia have adjusted well and show no behavioral or emotional problems according to their parents. Furthermore, children aged 6–18 years born with CDH seem to be socially competent and function well in school and during their leisure time, which was also confirmed by the parents own words when describing their children. However, when it comes to physical activities, less than half in the group of children born with CDH reach normal levels.
